# Proteomic responses of fruits to environmental stresses

**DOI:** 10.3389/fpls.2012.00311

**Published:** 2013-01-10

**Authors:** Zhulong Chan

**Affiliations:** Key Laboratory of Plant Germplasm Enhancement and Specialty Agriculture, Wuhan Botanical Garden, Chinese Academy of Sciences, Wuhan, Hubei, China

**Keywords:** proteomics, postharvest, fruit, pathogen, induced resistance, secretome

## Abstract

Fruits and vegetables are extremely susceptible to decay and easily lose commercial value after harvest. Different strategies have been developed to control postharvest decay and prevent quality deterioration during postharvest storage, including cold storage, controlled atmosphere (CA), and application of biotic and abiotic stimulus. In this review, mechanisms related to protein level responses of host side and pathogen side were characterized. Protein extraction protocols have been successfully developed for recalcitrant, low protein content fruit tissues. Comparative proteome profiling and functional analysis revealed that defense related proteins, energy metabolism, and antioxidant pathway played important roles in fruits in response to storage conditions and exogenous elicitor treatments. Secretome of pathogenic fungi has been well-investigated and the results indicated that hydrolytic enzymes were the key virulent factors for the pathogen infection. These protein level changes shed new light on interaction among fruits, pathogens, and environmental conditions. Potential postharvest strategies to reduce risk of fruit decay were further proposed based on currently available proteomic data.

## Introduction

Fruits and vegetables are highly perishable horticultural products, especially during the ripening and postharvest stages, when considerable losses due to microbiological diseases, disorders, transpiration, and senescence can occur. Although quality deterioration of fresh postharvest fruits and vegetables is the result of a number of different factors, microbial activity is by far the single most important one (Sommer, 1982). In the developed countries, approximately 10–30% of harvested fruits and vegetables is lost due to postharvest spoilage, and in the developing countries the losses are over 30–50% annually due to lacking sanitation and refrigeration (Salunkhe et al., 1991; Legard et al., 2000).

The fruit-pathogen interaction depends on mutual recognition. Studies have been performed using classical genetics, cell biology, and biochemistry, as well as high-throughput—omic techniques (Chan and Tian, 2005, 2006; Chan et al., 2007; Qin et al., 2007). As a powerful tool, proteomics approach has been widely used to identify global changes in structure and abundance of plant proteins in response to developmental and environmental signals (Chan et al., 2007; Shi et al., 2008). There has been an increasing trend in application of proteomic methods to detect fruit and vegetable physiological changes over the last few years (Rocco et al., 2006; Chan et al., 2008; Pedreschi et al., 2008), offering to the research community the opportunity to unravel complex sets of proteins.

## Development of proteomic profiling protocols for fruits and vegetables

Today, proteomics techniques, including high-resolution two dimensional electrophoresis (2-DE), in-gel proteolytic digestion of protein spots and protein identification by MS through database searches, are increasingly used in studies of animals and microorganisms (Antelmann et al., 1997; Qin et al., 2007), and have been used successfully in plant biology to study changes in protein level expression during development (Chang et al., 2000; Chan et al., 2007). However, plant cells contain many components that may interfere with protein extraction, separation, and purification (Granier, 1988). Fruit tissues are difficult to process through proteomic approach partly due to technical problems such as the low protein content of fruit and the presence of an array of compounds, for example, pigments, starch, polyphenols, polysaccharides, tannins, and organic acids that can cause a high degree of protein denaturation and inactivation (Clements, 1970).

**Figure 1:**
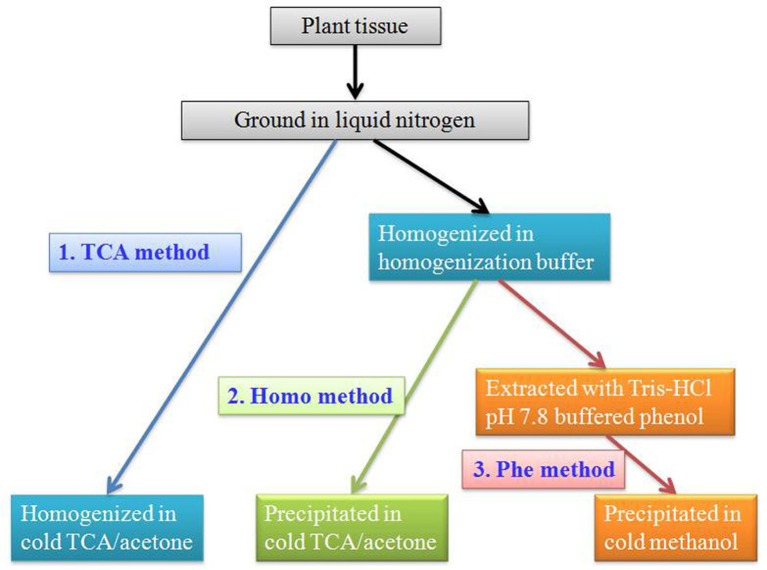
**Protein extraction protocols for fruits**.

For good reproducibility of 2-DE, sample preparation is a critical step. The extraction of high-quality protein from recalcitrant, low protein content fruit tissue is a challenge. Several protein extraction protocols suitable for 2-DE have been developed. Major soluble proteins of grapevine ripe berries have been extracted from six different cultivars using TCA-acetone solution (TCA method) (Sarry et al., 2004), resulting in three hundreds detected spots on the 2-DE map after colloidal blue staining. Chan and co-authors have developed two additional protocols suitable for fruit protein extraction (Chan et al., 2007, 2008). In one protocol, fruit tissues were homogenized in homogenization buffer containing 20 mM Tris-HCl (pH 7.5), 250 mM sucrose, 10 mM EGTA, 1 mM phenylmethanesulfonyl fluoride (PMSF), 1 mM dithiothreitol (DTT), and 1% Triton X-100 before TCA-acetone precipitation (Homo method) (Chan et al., 2007). In another protocol, extraction with an equal volume of Tris-HCl pH 7.8 buffered phenol was conducted after buffer homogenization but before methonal precipitation (Phe method) (Chan et al., 2008) (Figure 1). In comparison of these three protocols, Homo and Phe methods are much better than TCA method in fruit protein extraction. Reproducible 2-DE results with well-resolved polypeptide spots throughout the gel were achieved by Homo and Phe protein extraction protocols for a variety of fruits, including sweet cherry (*Prunus avivum*), peach (*Prunus persica*), apple (*Malus domestica*), mango (*Mangifera indica*), and jujube (*Ziziphus jujuba*) fruits. This method can also be applied as a general method to other plant tissues that are rich in interfering compounds (Wang et al., 2009a).

## Interaction between fruits and fungi

As mentioned above, many studies focused on the interactions between fruits and pathogens based on proteomics approach. However, the major problem is how to identify proteins from fruit-pathogen mixed samples. Therefore, few proteomic data are available for fruit (host) side and also for pathogen side *in vivo*. One possibility is that specific taxon should be used when mass spectrometry data were submitted to the website of Matrix Science for protein identification. After searching using both host taxon and pathogen taxon, we are able to tell where the proteins come from. Gray mold by *Botrytis cinerea* and green mold by *Penicillium expansum*, two major diseases caused enormous fruit decay and great economic losses, have been extensively studied by several research groups and part of the results were reviewed here.

### Botrytis cinerea

*B. cinerea* is a pathogenic filamentous fungus which infects more than 200 plant species in a variety of organs including fruit, flowers, and leaves (Williamson et al., 1995; Fernández-Acero et al., 2011). The host range for *B. cinerea* infection includes economically important crops such as tomato, berries, chickpeas, french beans, and cut flowers as well as many fruits. The fungus causes the gray mold disease resulting in significant crop losses under different production conditions. Gray mold occurs over a wide geographical area, in the open field, in greenhouses and even in storages at 0–10°C. *B. cinerea* is the principal cause of pre- and post-harvest disease in grapes, berries, tomatoes, and many other crops (Elad, 1997; Williamson et al., 2007). Proteomic analysis of three types of tomato fruit infected by *B. cinerea* revealed that 186 tomato proteins were identified in common among red ripe and red ripe-equivalent ripening inhibited (rin) mutant tomato fruit infected by *B. cinerea*. However, the limited infections by *B. cinerea* of mature green wild type fruit resulted in 25 and 33% fewer defense-related tomato proteins than in red and rin fruit, respectively (Shah et al., 2012).

The biology of *B. cinerea* has been studied extensively and the genome of the fungus has been sequenced (Elad et al., 2004; van Kan, 2006), including strain T4 (INRA/Genoscope: http://urgi.versailles.inra.fr/projects/Botrytis/) and strain B05.10 (Broad Institute, Massachusetts Institute of Technology, MIT, http://www.broad.mit.edu/annotation/genome/botrytis_cinerea/Home.html). *B. cinerea* secretes a battery of enzymes utilized for the degradation and consumption of the host plant. In 2006, Fernández-Acero et al. reported the first approach to the proteome analysis of *B. cinerea*. Most of the identified spots may play a crucial role as pathogenicity or virulence factors, including some housekeeping enzymes, such as malate and glyceraldehyde dehydrogenases. In an attempt to identify putative fungal virulence factors, protein profile from two *B. cinerea* strains differing in virulence and toxin production were compared. Twenty seven protein spots were identified and a significant number of spots were identified as malate dehydrogenase and glyceraldehyde-3-phosphate dehydrogenase, which could be ascribed to differences in virulence between strains (Fernández-Acero et al., 2007). Shotgun proteomics was successfully used to identify the secretome of *B. cinerea* grown in three culture conditions which differed by the carbon nutrients provided. A total of 126 proteins secreted by *B. cinerea* were identified, 13 of which were identified as pectinases, which play an important role in cell wall degradation and successful invasion (Shah et al., 2009). Furthermore, the effect of ambient pH on secretome of *B. cinerea* strain B05.10 was investigated with a comparative proteomic method. The results indicated that distinct differences in secretome of *B. cinerea* were found between pH 4 and 6 treatments, and 47 differential spots, corresponding to 21 unique proteins, were identified. At pH 4, more proteins related to proteolysis were induced, whereas most of up-accumulated proteins were cell wall degrading enzymes at pH 6 (Li et al., 2012).

### Penicillium expansum

*P. expansum*, another widespread filamentous fungus, is a major causative agent of fruit decay with great economic losses. This strain is also of potential public health concern, because it produces toxic secondary metabolites, including patulin, citrinin, and chaetoglobosins (Andersen et al., 2004).

In a study to compare the cellular and extracellular proteomes of *P. expansum*, the results showed that several proteins related to stress response (glutathione S-transferase, catalase, and heat shock protein 60) and basic metabolism (glyceraldehyde-3-phosphate dehydrogenase, dihydroxy-acid dehydratase, and arginase) were identified in the cellular proteome. Catalase and glutathione S-transferase, the two antioxidant enzymes, exhibited reduced levels of expression upon exposure to borate, which affects the virulence of the fungal pathogen. The extracellular proteome of *P. expansum* under stress condition with reduced virulence showed that the expression of three protein spots were repressed in the presence of borate and identified as the same hydrolytic enzyme, polygalacturonase (Qin et al., 2007).

Exogenous environmental conditions greatly affect infection of pathogens, including temperature, humility, pH value, and oxidative status. H_2_O_2_ is reported to have a direct antimicrobial effect and be involved in defense reactions activated in plant tissues upon pathogen attack (Mellersh et al., 2002). Plasma membrane damage was not the main reason for H_2_O_2_-induced death of the fungal pathogen. Proteomic analysis of the changes of total cellular proteins in *P. expansum* showed that a large proportion of the differentially expressed proteins appeared to be of mitochondrial origin, implying that mitochondria may be involved in this process. Further mitochondrial sub-proteomic analysis characterized a set of mitochondrial proteins, including respiratory chain complexes I and III, F_1_F_0_ ATP synthase, and mitochondrial phosphate carrier protein which might be associated with fungal death caused by H_2_O_2_ (Qin et al., 2011). The pH value, as one of the most important environmental parameters, has critical influence on spore germination of *P. expansum*. Spore germination of *P. expansum* was obviously inhibited at pH 2.0 and 8.0. Comparative proteomics analysis revealed that 17 proteins involved in protein synthesis and folding were mainly down-regulated at pH 2.0 and 8.0. These findings indicated that impairing synthesis and folding of proteins might be one of the main reasons account for ambient pH effect on spore germination of *P. expansum* (Li et al., 2010).

## Induced resistance (IR) of fruits

**Figure 2:**
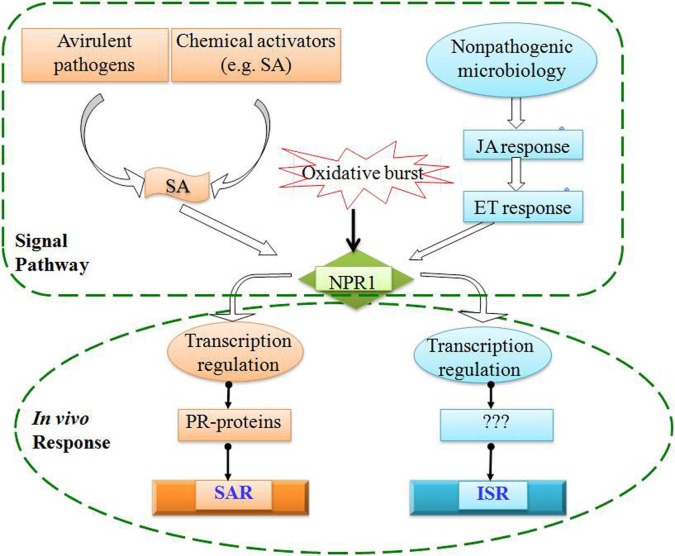
**Signal transduction pathways for systemic acquired resistance (SAR) and induced systemic resistance (ISR)**.

Traditionally, postharvest disease is often controlled by the application of synthetic fungicides (Eckert and Ogawa, 1988). However, chemical protection is discouraged due to problems related to fungicide toxicity, development of fungicide resistance by pathogens, and potential harmful effects on the environment and human health, alternatives to synthetic chemicals have been proposed (Eckert et al., 1994; Tian and Fan, 2000; Elad et al., 2004). The use of biologically based fungicides in conjunction with induced resistance (IR) was suggested as a feasible approach for reducing postharvest disease in harvested fruits and vegetables (Cook et al., 1999; Tian et al., 2001). IR is a plastic response, which diverts carbon and nitrogen resources from plant growth and reproduction to provide a long lasting and systemic resistance to a broad spectrum of pathogens and pests (Linda, 2001). Two types of IR are well-characterized. Systemic acquired resistance (SAR) is an active defense initiated by infection with certain necrotizing pathogens and confers resistance to secondary infection. SAR is effective against a broad-spectrum of pathogens including viruses, bacteria, fungi, and oomycetes (Ryals et al., 1996; Sticher et al., 1997). Inhibition of salicylic acid (SA) accumulation or biosynthesis impairs SAR (Gaffney et al., 1993). Induced systemic resistance (ISR) resembles SAR but is induced by root colonization of specific strains of non-pathogenic plant growth-promoting rhizobacteria in contrast to SAR that is induced by necrotizing pathogens. Unlike SAR, ISR is dependent on jasmonate and ethylene, independent on SA and not associated with *PR* gene expression (van Loon, 1997). Molecularly, both SAR and ISR in Arabidopsis are intertwined through *NPR1* gene (Figure 2).

In fruits, IR can be triggered by microbial biological agents (non-pathogens, avirulent forms of pathogens), physical agents (curing, γ-radiation, hot water brushing and UV-C light), certain chemical agents [DL-3-amino butyric acid (BABA), 1,2,3-benzothiadiazole-7-carbothioic acid S-methyl ester (ASM), salicylic acid (SA), ethylene, harpin, 2,6-dichloroisonicotinic acid, jasmonic acid (JA), methyl jasmonate (MJ), Oxalic acid (OA), potassium and phosphates], and natural compounds (Chitosan and Margosan-O) (Tian and Chan, 2004). In many experiments, IR holds promise as a new technology for the control of postharvest diseases and has been proven to be effective in the laboratory and in a few field cases (Droby et al., 2001; El-Ghaouth et al., 2003; Chan et al., 2008). Mechanisms of IR have been well-characterized from cell structure, physiological, and biochemical changes. Proteomic studies shed a light on molecular changes of IR.

### Microbial biological agents

In recent years, considerable attention has been placed on postharvest application of antagonists for the inhibition of plant disease because of concerns about the application of synthetic chemicals. Utilization of antagonistic yeasts as an alternative appears to be a promising technology (Droby et al., 2001; El-Ghaouth et al., 2003; Chan and Tian, 2006). Some antagonist-based products are commercially available and others are currently under varying degrees of development (Castoria et al., 2001).

Several mechanisms have been reported to play a significant role in the biocontrol activity of antagonistic yeasts, including direct interaction between antagonistic yeasts and pathogens (El-Ghaouth et al., 2003; Chan and Tian, 2005) and IR of fruit tissues by antagonistic yeasts (Droby et al., 2001; Chan and Tian, 2006; Chan et al., 2007). Significant changes in polyphenoloxidase (PPO), peroxidase (POD), catalase (CAT), superoxide dismutase (SOD), and phenylalanine ammonia-lyase (PAL) activities were found to be involved in the IR after antagonistic yeasts treatment (Fan and Tian, 2000; Qin et al., 2002; Chan and Tian, 2006). In harvested peach fruit, 19 proteins were identified using quadrupole time of-flight tandem mass spectrometer after treatment with antagonistic *Pichia membranefaciens*, including antioxidant related proteins, stress responsive proteins and proteins involved in energy pathways (Chan et al., 2007). These results suggested that antioxidant and PR proteins, as well as enzymes associated with sugar metabolism, were involved in resistance of peach fruit induced by *P. membranefaciens*.

### Heat treatment

Pre-storage heat treatment is a promising postharvest method for reducing disease incidence and severity (Terry and Joyce, 2004; Schirra et al., 2011). Pre- and post-harvest heat treatments increased the resistance of cherry tomato (Zhao et al., 2009), bamboo shoots (Luo et al., 2012), fresh cut broccoli (Moreira Mdel et al., 2011), strawberry (Marquenie et al., 2002; Villa-Rojas et al., 2011), lemon (Nafussi et al., 2001) against postharvest decay, and reduced the development of green mold on citrus fruit (Schirra et al., 2008a), blue mold on pear (Schirra et al., 2008b), and peach (Zhang et al., 2007).

Based on the 2D-DIGE analysis, 52 proteins were differentially expressed between peach fruits exposed to heat treatment or after transfer to 20°C, vs. fruits kept at 20°C. Among identified spots, a large number (93%) have been proposed to play a role in plant metabolism such as the defense and stress response, cytoskeleton organization, primary metabolism, transcription and translation regulation, and protein storage and catabolism. The category of proteins participating in biotic or abiotic stress responses was the one with the most proteins differentially expressed. Additionally, one third of the identified proteins corresponded to the large family of HSPs and exhibited molecular masses <20 kDa, indicating that the induction of small HSPs in heated peach may participate in the acquisition of tolerance against some chilling injury symptoms (Lara et al., 2009). Another study demonstrated that among the thirty protein spots in peach fruit induced by heat treatment, 43% were related to stress response, 17% to cell structure, 13% to protein fate, 7% to glycolytic pathway, 3% to ripening and senescence, and 17% to unclassified (Zhang et al., 2011). The commonly induced proteins from both groups included ascorbate peroxidase, heat shock proteins, and allergen proteins, indicating these proteins are heat inducible proteins in peach fruit.

In citrus fruit, heat treatment induces defense mechanisms and triggers physiological responses to withstand stressful conditions during storage. Functional classification of twenty eight differentially expressed proteins showed that the main affected categories were “Cell rescue, defense, and virulence” and “Metabolism.” Activity of antioxidant enzymes was extensively changed upon heat treatment, including SOD, POD and alcohol dehydrogenase (Perotti et al., 2011).

### Chemical agents

Certain chemicals, including SA and OA, emerged as potentially effective agents to IR in fruits. SA has emerged as a key signaling component involved in the activation of certain plant defense responses (Durner et al., 1997). Exogenous application of SA protects plants against certain pathogens and activates SAR in a wide variety of plant species, including harvested fruit (van Loon, 1997; Yao and Tian, 2005). SA treatment IR to MYMIV infection in *Vigna mungo*. Twenty-nine proteins identified by MALDI-TOF/TOF, predicted to be involved in stress responses, metabolism, photosynthesis, transport, and signal transduction, showed increased abundance upon SA treatment. SA treatment stimulated SOD and POD activity and inhibited CAT activity thus preventing ROS mediated damage (Kundu et al., 2011). In harvested peach fruit, thirteen SA induced proteins were identified and the functions of these proteins were mainly involved in antioxidant and energy pathway (Chan et al., 2007). Pre-harvest application of SA solution enhanced the resistance of sweet cherry fruits against *P. expansum*, resulting in lower disease incidences and smaller lesion diameters, especially at earlier maturity stage. Totally 13 and 28 proteins were identified after SA treatment at earlier (A) and later (B) maturity stage, respectively. Antioxidant proteins and pathogenesis related-proteins were identified at both A and B stages, while heat shock proteins and dehydrogenases involved in glycolysis and tricarboxylic acid cycle were only detected at B stage (Chan et al., 2008). All these results indicated that antioxidant related proteins as well as proteins involved energy metabolism play key roles in SA induced resistance in fruits.

OA is an organic acid distributing widely in various organisms, especially in plants (Franceschi and Nakata, 2005). Recent studies revealed that OA might play important roles in systemic resistance, stress response, programmed cell death and redox homeostasis in plant (Guo et al., 2005; Kim et al., 2008). Recent studies showed that application of OA extend postharvest and shelf life in litchi (Zheng and Tian, 2006), mango (Zheng et al., 2007a), peach (Zheng et al., 2007b), jujube (Wang et al., 2009b), and plum (Wu et al., 2011) fruits. The application of oxalic acid reduced ethylene production and delayed softening of plum fruit. During storage or shelf-life, flesh reddening and anthocyanin synthesis were significantly inhibited in oxalic acid-treated plum fruit, accompanied with decreased PAL activity (Wu et al., 2011). In jujube fruit, application of OA at the concentration of 5 mM could delay fruit senescence by reducing ethylene production, repressing fruit reddening and reducing alcohol content, which consequently increased fruit resistance against blue mold caused by *P. expansum*. A total of 25 differentially expressed proteins were identified after OA treatment. The activity of alcohol dehydrogenase 1 and 1-aminocyclopropane-1-carboxylic acid synthase was repressed, while the abundances of three photosynthesis-related proteins, a cystathionine β-synthase domain-containing protein and three proteins related to the defense/stress response were up-regulated by OA. These results indicated that OA treatment might affect ethanol and ethylene metabolism, resulting in delaying senescence, and increase resistance of jujube fruits against fungal pathogens (Wang et al., 2009b).

## Proteome-level responses of fruits to storage environments

### Cold storage

Cold storage is one of the most frequently employed methods to delay fruit senescence and maintain fruit quality during post-harvest storage. Generally speaking, fruits taste good with a total soluble sugar/total organic acid ratio over 8 and low temperature (LT) is applied in inhibiting the ratio increase (Yun et al., 2012). Under LT condition, a total of 108 differentially accumulated protein spots in citrus were analyzed by MALDI-TOF MS/MS, and 63 spots were successfully identified on the basis of tryptic peptide sequences. The results showed that metabolism pathway and stress response related categories were considerably enriched, including sugars and polysaccharide metabolism, secondary metabolism, protein destination and storage, and response to stimulus (Yun et al., 2012). Moreover, fruit maintains low accumulation of ROS, low level of membrane lipid peroxidation, greater flesh firmness and higher concentrations of organic acids and, vitamin levels (Kan et al., 2011; Yun et al., 2012). These results indicated that these processes play a leading role in the maintenance of fruit quality and delaying of senescence at LT.

Interestingly, peach fruit is less prone to chilling injury when stored at 0°C than at 5°C (Zhang et al., 2010). Further study using proteomics approach indicated that several membrane stability related proteins were enhanced, while proteins related to phenolic compounds metabolization were repressed in peach fruit at 0°C relative to 5°C. Other proteins involved in sugar metabolism and energy pathways were decreased at 0°C, resulting in the lower assumption of sugars which has several beneficial effects in protecting plants against stresses (Zhang et al., 2010). When stored at LTs, 55 proteins from bell pepper (*Capsicum annuum*) fruits were identified as having differential abundances. Redox and carbohydrate metabolism were the two major functional classes found in a proteomic study (Sánchez-Bel et al., 2012).

Dehydrin is known as a group 2 late embryogenesis abundant (LEA) proteins, one of several ubiquitous water-stress-responsive proteins in plants (Close, 1997). Several individual groups identified dehydrin protein in peach and citrus fruit stored at LT through traditional 2D-PAGE and difference gel electrophoresis (DIGE) (Nilo et al., 2010; Yun et al., 2010, 2012; Zhang et al., 2010). Other commonly identified proteins by these groups included heat shock protein and dehydrogenase which were well-known to play a key role in plant stress responses (Giannino et al., 2004; Posner et al., 2012). In nectarine fruits stored at LT, four differentially expressed proteins were characterized as allergens which can provide some form of protection to fruits during periods of stress (Pedreschi et al., 2007; Giraldo et al., 2012).

### Controlled atmosphere (CA)

Fruit are often stored under controlled atmosphere (CA) conditions with low-O_2_ and high-CO_2_ at LT to reduce respiration, ethylene production rates and action, delay ripening and senescence, as well as to reduce the growth of pathogens (Kader, 2002). When loquat (*Eriobotrya japonica* Lindl.) fruit were stored under different storage conditions, CA with 10% O_2_ + 1%CO_2_ was more effective in reducing fruit decay. Loquat fruit could be stored in this CA condition at 1°C for more than 50 d with normal flavor and low decay index of about 7%. CA conditions were more effective in reducing the activities of PPO and oxidative stress compared to other treatments (Ding et al., 2006). The same results were achieved that CA conditions were more effective in reducing total phenol content, delaying anthocyanidin decomposition, preventing pericarp browning, and decreasing Litchi (*Litchi chinensis* Sonn. cv Heiye) fruit decay in comparison with other treatments (Tian et al., 2005a). Proteomic analysis revealed that several proteins were significantly changed in pear during CA conditions. Triosephosphate isomerase, a key enzyme of the energy metabolism was up-regulated under browning-inducing conditions. ACC oxidase involved in ethylene biosynthesis and the major allergen Pyrc 1 were clearly down-regulated under low oxygen or high carbon dioxide concentrations. Stress responsive proteins, like the chaperone molecule HSP70, were also down-regulated as the oxygen concentration diminished (Pedreschi et al., 2008).

## Proteome-level changes during fruit development and ripening

Fruit ripening is a developmental complex process which occurs in higher plants and involves a number of stages displayed from immature to mature fruits that depend on the plant species and the environmental conditions (Palma et al., 2011). The pathways involved in the processes of fruit development and ripening are exclusive for plants and vary between species. Based on ethylene production, two types of fruits were characterized, i.e., either climateric or non-climateric. Climacteric fruits, such as tomato, papaya, peach, apple, pear, banana, plum, and melon, are characterized by a dramatic increase in ethylene production, which is responsible for the typical respiratory burst during ripening, and the activation of many biochemical steps (Barry and Giovannoni, 2007; Palma et al., 2011). On the other hand, ripening of non-climateric fruits such as pepper, citrus, orange, cucumber, grape, cherry, and strawberry is ethylene-independent (Palma et al., 2011).

### Climateric fruits

In young tomato fruit, intensity of proteins involved in amino acid metabolism and protein synthesis increased during the early development (cell division) stage. During the later development (cell expansion) stage, proteins functioning in photosynthesis and cell wall formation transiently increased. In contrast, many proteins related to C compounds and carbohydrate metabolism or oxidative processes were up-regulated during fruit development (Faurobert et al., 2007). In papaya, six main categories, including cell wall, ethylene biosynthesis, climacteric respiratory burst, stress response, synthesis of carotenoid precursors, and chromoplast differentiation were found to be related to fruit ripening using 2DE-DIGE (Nogueira et al., 2012). Based on traditional 2D-PAGE, several cell wall degrading enzymes related to fruit ripening were identified in papaya fruit (Huerta-Ocampo et al., 2012). In peach fruit, the functions of 30 identified proteins were involved in primary metabolism (e.g., C-compounds, carbohydrates, organic acids, and amino acids) and in ethylene biosynthesis as well as proteins involved in secondary metabolism and responses to stress (Prinsi et al., 2011). A further study to characterized the protein accumulation patterns in firm and soft fruit of three peach and two nectarine melting flesh varieties revealed that 164 of the 621 protein spots analyzed displayed a differential accumulation associated with the softening process. Among them, only 14 proteins changed their accumulation in all the varieties assessed, including proteins mostly involved in carbohydrates and cell wall metabolism as well as fruit senescence (Nilo et al., 2012). Moreover, in kiwifruit, which displays climacteric behavior at temperatures above 20°C (Antunes, 2007), proteomic analysis using 1D-SDS-PAGE and mass spectrometry identified 102 kiwifruit proteins during ripening, which are mainly involved in energy, protein metabolism, defense, and cell structure. Ripening induced protein carbonylation in kiwifruit but this effect was depressed by ozone (Minas et al., 2012).

### Non-climateric fruits

The proteins identified as differentially accumulated during ripening of strawberry fruit are involved in a wide range of biological processes such as energy and carbon metabolisms, secondary metabolism/biosynthesis of cellular components, cellular organization, communication and signal transduction, protein metabolism, stress response, and transcription. Most of identified proteins showed a regular increase in spot volume from the immature to the mature stage indicating they are progressively involved during ripening (Bianco et al., 2009). Grape is another non-climateric important crop species for human nutrition and agricultural economy. The proteomic analysis using grape skin tissue revealed that the most relevant changes in protein expression occurred in the first weeks of ripening. Many of these variations were related to proteins involved in responses to stress, glycolysis and gluconeogenesis, C-compounds and carbohydrate metabolism, and amino acid metabolism (Negri et al., 2008). Another study revealed that proteins involved in photosynthesis, carbohydrate metabolisms, and stress response are identified as being enriched at the beginning of color-change. The end of color-change is characterized by the enrichment of proteins involved in anthocyanin synthesis and, at harvest, the dominant proteins are involved in defense mechanisms. In particular, the abundance of different chitinase and beta-1,3-glucanase isoforms increased as the berry ripens, indicating these enzymes were involved in softening during fruit ripening (Deytieux et al., 2007).

## Tools for fruit proteome profiling

Traditional large scale proteomic analysis usually relies on 2D gel electrophoresis (2DE), i.e., separation by 2DE, protein spot digestion with a protease, and MS identification. However, gel electrophoresis is poorly compatible with high-throughput MS analysis. More recently, gel free technology has been well-developed, which includes steps of protease digestion of the protein mix, LC separation of peptides, and MS identification. Though the 2DE is still the most commonly used method, gel free based protein separation approaches, like MudPIT (multidimensional protein identification technology), DIGE (2D-difference gel electrophoresis), iTRAQ (Isobaric tag for relative and absolute quantitation), SILAC (stable isotope labeling by amino acids in cell culture), ICAT (isotope coded affinity tags), and LOPIT (localization of organelle proteins by isotope tagging), are potentially powerful tools for fruit proteomes analyses.

2D-DIGE is a powerful technique for quantitatively comparing different samples labeled with different dyes. Using this technology, 37 proteins showing different abundance during papaya fruit ripening were characterized and submitted to MS analysis (Nogueira et al., 2012). In addition, approximately 2500 protein spots were successfully resolved from ripening strawberry fruit 2D-DIGE gel (Bianco et al., 2009). In iTRAQ, peptides derived from each sample are derivatized with amine-specific isobaric tags which are indistinguishable by MS but exhibit MS/MS signature ions. A total of 1664 proteins were identified from orange by the iTRAQ technique (Ai et al., 2012). Researchers also successfully identified genetically engineered tomato using iTRAQ and MudPIT methods (Robertson et al., 2012). These non-gel based approaches are complementary to gel-based ones and together the different techniques allow for improved proteomic coverage (Quirino et al., 2010).

## Concluding remarks and future prospect

**Figure 3:**
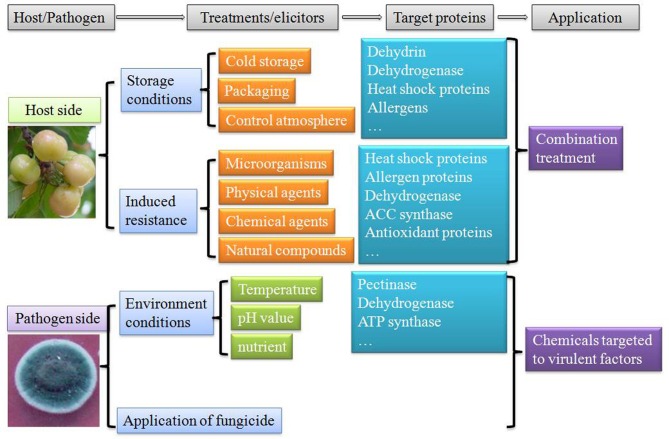
**Approaches to control postharvest decay and mechanisms involved based on proteomic study**.

Proteomics approach is not only a powerful tool to dissect fundamental level changes, but for selection of appropriate markers (proteins) so as to be able to detect metabolic disorders in harvested fruits at an early stage and identify virulent factors involved in pathogen infection. Based on proteomic studies, proteins involved in different metabolic pathways in fruit were activated after postharvest treatments. Therefore, to reduce postharvest decay and thus minimize ecological losses, biologists should firstly pay more attentions to combination treatments. Several pioneering works have been carried out to control postharvest diseases by combining different approaches (Tian et al., 2005b; Palou et al., 2007; Sivakumar et al., 2008; Wang et al., 2011). Combination treatments showed improved control efficiency to reduce fruit disorder and decay, and maintain fruit quality during postharvest storage and transportation. Moreover, proteomic approach from the pathogen side also revealed that virulent factors play key roles during pathogen infection. Specific medicines targeted to these proteins could be designed to inhibit the development and growth of pathogens (Figure 3). Through these two strategies the biologists were able to more effectively protect the fruit from the infection of postharvest pathogens. Further researches combining proteomic, transcriptomic, and metabolic approaches are also needed to characterize mechanisms involved in interactions among fruits, pathogens and environmental conditions.

### Conflict of interest statement

The author declares that the research was conducted in the absence of any commercial or financial relationships that could be construed as a potential conflict of interest.
